# Is your drinking-water safe? A rotavirus outbreak linked to water refilling stations in the Philippines, 2016

**DOI:** 10.5365/wpsar.2017.8.1.007

**Published:** 2019-02-20

**Authors:** Niño D Rebato, Vikki Carr D de los Reyes, Ma Nemia L Sucaldito, Gretchen R Marin

**Affiliations:** a Department of Health, Philippines.

## Abstract

**Introduction:**

In April 2016, the Department of Health in Zamboanga Peninsula reported an increase in the number of acute gastroenteritis cases reported from Zamboanga City. An epidemiologic investigation was conducted to verify the existence of an outbreak, determine source/mode of transmission and recommend control measures.

**Methods:**

A line list of cases was compiled from the 11 hospitals within Zamboanga City and a case-series study was conducted. Suspected cases were any persons from Zamboanga City who had three or more episodes of acute diarrhoea within 24 hours from 15 March to 29 May 2016. Confirmed cases were suspected cases with active symptoms during the investigation who had a stool sample collected with rotavirus detected. Water samples were also collected for viral detection.

**Results:**

There were 2936 suspected cases with 22 deaths (case fatality rate: 0.75%), an age range of 8 days to 89 years (median: 2 years), with those aged less than 5 years the most affected age group (1903/2936, 65%). The majority were males (1549/2936, 53%). From the 138 active case patients included in the case-series study, the majority reported contact with a family member who had diarrhoea (89/138, 64%) and using water refilling stations as their major source of drinking-water (88/134, 64%). Of the 93 stool specimens collected, 56 (60%) were positive for rotavirus. Five samples from water refilling stations where case patients reported collecting drinking-water were all positive for rotavirus.

**Discussion:**

Strict regulation of water refilling stations and boiling drinking-water in households were implemented, immediately controlling the outbreak. After complying with all the requirements set by the Department of Health, a water safety certificate was awarded to Zamboanga City in September 2018.

## Introduction

On 6 April 2016, the Regional Epidemiology and Surveillance Unit of the Department of Health in Zamboanga Peninsula reported an increase in acute gastroenteritis cases in Zamboanga City to the national event-based surveillance system. Zamboanga City is a highly urbanized city located in Mindanao in the southern Philippines with a population of 861 799 people. (*1*) It is the sixth most populous and third largest city in the Philippines. (*2*) Water production within Zamboanga City Water District serves only 48% of the total population with most people relying on water refilling stations. These water

According to Zamboanga City Health Office, the city had a rotavirus outbreak in 2010 due to water contamination, affecting around 500 individuals. In 2012, rotavirus vaccine was added to the national vaccination schedule of all infants aged between 1.5 and 3.5 months. According to Zamboanga City Health Office, the immunization coverage in Zamboanga City is around 30%, far from the national target of 90%. (*3*)

The Epidemiology Bureau of the Department of Health sent a team from 28 April to 2 May 2016 to conduct an epidemiologic investigation to verify the existence of an outbreak, determine the cause, identify the likely source and mode of transmission and recommend control and prevention measures.

## Methods

### Case-series study

A line list of suspected cases was constructed from records on suspected case patients admitted to the 11 hospitals within Zamboanga City. A suspected case was defined as any previously well individual in Zamboanga City who had three or more watery stools per day from 15 March to 29 May 2016.

A case-series study was then conducted for those suspected cases that were still symptomatic and admitted to hospital at the time the investigation team was present (active cases). These active case patients were interviewed for information on demographics, exposure history, medical history and other relevant information. Stool samples were collected from these active case patients with a confirmed case defined as a suspected case that had rotavirus detected in stool sample.

Data analysis was performed using Microsoft Excel 2013.

### Environmental investigation

The Philippine drinking-water standards require all water sources to adhere to standard parameters and values for drinking-water quality. There should be no *Escherichia coli;* coliform or other bacteria present in every 100 ml water sample should not exceed the permissible level of biological organisms, organic and inorganic constituents (antimony, arsenic, etc.); and the chemical, disinfectant, disinfectant by-products and radiological constituents should be within standard values. (*4*)

Sixty-six water sources consisting of 39 water refilling stations, 17 water district and 10 deep wells in Zamboanga City were inspected by the city sanitation office to determine possible cross-contamination and non-compliance to the Philippine drinking-water standards. (*4*)

### Laboratory examination

Stool specimens from active case patients were collected and sent to the Research Institute for Tropical Medicine (RITM) in Manila for rotavirus, norovirus and adenovirus detection using reverse transcriptase polymerase chain reaction (RT–PCR). To rule out other potential causes of the outbreak, rectal swab specimens were also tested for bacterial pathogens according to RITM standard procedures and previously published methods. (*5*)

Water samples were tested for bacterial analysis using the Colilert (IDEXX Laboratories, Inc., Westbrook, Maine, USA) rapid test. RT–PCR and conventional PCR were used for detection of pathogen based on the World Health Organization’s manual for rotavirus detection. (*6*)

## Results

### Suspected cases

**Fig. 1:**
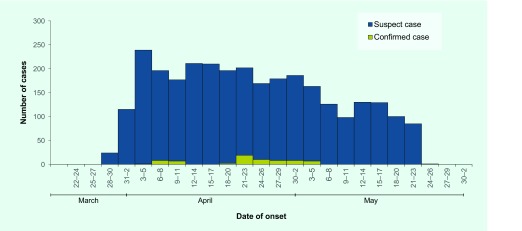
Diarrhoea cases by date of onset and case category, Zamboanga City, 28 March to 29 May 2016 (*n* = 2936)

A total of 2936 suspected cases were identified with onset dates from 28 March 2016. There was a peak of cases on 3–5 April 2016 (**Fig. 1**). The age of all suspected cases ranged from 8 days to 89 years (median: 2 years), with the majority of suspected cases aged less than 5 years (1903/2936, 65%). Just over half the suspected cases were male (1549/2936, 53%). There were 22 deaths reported, giving a case fatality rate of 0.75%. The age of fatal cases ranged from 2 months to 50 years (median: 11 months) and 13 (59%) were male.

### Case series

There were 138 active case patients included in the case series. Eighty-eight (64%) were aged less than 5 years. All had diarrhoea; others reported vomiting (112/138, 81%), abdominal pain (84/138, 61%) and fever (83/138, 60%).

The majority of active case patients reported close contact with family member who had diarrhoea before their illness onset (89/138, 64%). The major source of drinking-water reported among these case patients was from water refilling stations (88/138, 64%; only 44/138 or 32% reported that they drank boiled water. Before eating and after using toilet, 53% (73/138) reported using hand sanitizer and 62% (86/138) reported using detergent or bath soap regularly (**Table 1**).

**Table 1:** Profile of active rotavirus cases, Zamboanga City, 28 April to 2 May 2016 (*n* = 138)

Factors	< 5 years *n*(%)	≥ 5 years *n*(%)	Total *n*(%)
**Total**	**88 (100%)**	**50 (100%)**	**138 (100%)**
**Sex**			
**Male**	**48 (55%)**	**21 (42%)**	**69 (50%)**
**Female**	**40 (45%)**	**29 (58%)**	**69 (50%)**
**Symptoms**			
**Diarrhoea**	**88 (100%)**	**50 (100%)**	**138 (100%)**
**Vomiting**	**80 (91%)**	**32 (64%)**	**112 (81%)**
**Abdominal pain**	**50 (57%)**	**34 (68%)**	**84 (61%)**
**Dehydration**	**56 (64%)**	**37 (74%)**	**93 (67%)**
**Fever**	**58 (66%)**	**25 (50%)**	**83 (60%)**
**Hospitalized**	**88 (100%)**	**50 (100%)**	**138 (100%)**
**Close contact with sick family member**	**55 (55%)**	**34 (68%)**	**89 (64%)**
**Drinking-water source from water refilling stations**	**74 (84%)**	**14 (28%)**	**88 (64%)**
**Boil drinking-water**	**31 (35%)**	**13 (26%)**	**44 (32%)**
**Use hand sanitizer**	**45 (51%)**	**28 (56%)**	**73 (53%)**
**Use soap after toilet use**	**56 (64%)**	**30 (60%)**	**86 (62%)**

### Environmental survey

There were more than 200 water refilling stations in Zamboanga City, but only 125 had a sanitary permit. Non-compliant establishments were issued closure orders by the City Health Office due to possible contamination. Violations of the Philippine drinking-water standards were observed in some establishments due to presence of bacteria in water sample and biological organisms more than the permissible limit in every 100 ml sample.

Five water distribution pipes from Zamboanga City Water District were inspected. Water handlers were mostly children. Hand pumps are attached to the water distribution pipes to add pressure to the faucet. Pipes are also submerged in the sewers. These sources also did not meet the water-quality standards and were immediately rehabilitated.

### Laboratory examination

There were 93 stool specimens from active case patients submitted to RITM for viral detection with 56 (60%) positive for rotavirus. Six case patients (6%) had co-infection of rotavirus and adenovirus, and three (3%) had co-infection of rotavirus and norovirus. All specimens were negative for any bacterial pathogens.

Of the 66 water samples, excess total coliforms were detected in 19/39 (49%) water refilling stations, 9/17 (53%) Zamboanga City Water District samples and 4/10 (40%) samples from deep wells. *Escherichia coli* were present in water samples collected from water refilling stations (4/39, 10%), Zamboanga City Water District (2/17, 12%) and deep wells (5/10, 50%).

Of the 15 water samples sent to RITM for rotavirus confirmation (five each from water refilling stations, the Zamboanga City Water District and deep wells) only the five refilling-station samples were positive for rotavirus RNA.

## Discussion

The evidence collected during this outbreak investigation suggests that rotavirus was the cause of this outbreak. Sixty per cent of cases that were laboratory tested were positive for rotavirus; water samples taken from water refilling stations, the most commonly reported water source used by active case patients, were also positive for rotavirus. The symptoms reported were consistent with rotavirus, (*7*) as was the age distribution with most cases aged less than 5 years. Rotavirus was the most common cause of diarrhoeal deaths globally in children under 5 years old, accounting for 215 000 child deaths in 2013 based on the estimates of the World Health Organization. (*8*) This underscores the need for prevention in this particular age group.

Rotavirus is the major cause of hospital admissions, emergency department visits and clinic visits in the Philippines among infants with diarrhoea. (*9*) Rotavirus vaccine was introduced in 2012 as part of the Expanded Program on Immunization, but because of inadequate supply, some areas, including Zamboanga City, have not received the vaccine since 2014. Vaccination could help prevent rotavirus outbreaks.

Rotavirus infection can easily spread from an infected person to another by close contact. (*7*) Most case patients were exposed first to a family member with vomiting and diarrhoea before onset of their symptoms. Rotavirus is highly communicable; a small infectious dose of less than 100 virus particles can cause the fast spread of the illness. (*10*)

The majority of active case patients reported obtaining their drinking-water from water refilling stations. This was the first rotavirus outbreak in the Philippines with viral isolation of rotavirus from water refilling stations. The presence of rotaviruses in drinking-water has been reported in several studies (*11*, *12*) and has been the source of several epidemics originating from contaminated water. (*13*, *14*) In parallel with person-to-person contamination, drinking-water might play a role in the occurrence of sporadic cases. This, and the fact that many of the water refilling stations did not have sanitary permits, emphasize the need for routine inspections and water testing of water refilling stations.

RITM’s protocol (*5*) indicates that only stool specimens from cases whose onset of diarrhoea was three days before the specimen collection date could be tested. Because specimen collection was only done during the time of field investigation, cases that fell outside the collection period were not sampled, hence, only 93/2936 or 3% of stool specimens were collected. This sampling method could overestimate sample positivity. However, the higher prevalence of rotavirus in younger children demonstrated by this study is similar to other rotavirus investigations in other settings. (*12*-*14*)

Risk factors for this outbreak were not statistically tested due to the descriptive design of the study. Also, we were not able to elicit information on the rotavirus vaccination history of the active case patients interviewed as it was not included in our questionnaire. This is a limitation of the study, although it is expected that the vaccination rate would be low based on reported vaccination rates in the city.

In May 2016, as a result of this investigation, the government of Zamboanga City advised households to boil drinking-water and also created a technical working group focusing on water safety with emphasis on the strict regulation of water refilling stations to control the outbreak. Multiagency activities were conducted to come up with the city water safety plan, and in September 2018, more than two years after the outbreak, a water safety certificate was awarded to Zamboanga City after complying with all the requirements set by the Department of Health. No further outbreaks of rotavirus have been reported in Zamboanga. To prevent outbreaks of this magnitude in the future, these water safety measures need to be sustained and continued.
